# Effects of yoga compared with health promotion on health-related quality of life in adults with post-COVID-19 condition: protocol for a randomised controlled trial

**DOI:** 10.1136/bmjopen-2024-085525

**Published:** 2024-09-12

**Authors:** Mikaela Brodén, Paul Welfordsson, Maria Niemi, Vinod Diwan, Komal Shah, Vijayakumar Pattanadara, Mats Hallgren

**Affiliations:** 1 Department of Global Public Health, Karolinska Institutet, Stockholm, Sweden; 2 Indian Institute of Public Health, Public Health Foundation of India, Gandhinagar, Gujarat, India; 3 Department of Integrative Medicine, Sduaher, Kolar, Karnataka, India; 4 School of Exercise and Nutrition Sciences, Deakin University, Melbourne, Victoria, Australia

**Keywords:** Randomized Controlled Trial, Post-Acute COVID-19 Syndrome, Quality of Life, MENTAL HEALTH, Fatigue, PUBLIC HEALTH

## Abstract

**Introduction:**

Post-COVID-19 condition (post COVID, also known as long COVID) is a global public health issue estimated to affect over 100 million people. Common symptoms include fatigue, dyspnoea and cognitive dysfunction (‘brain fog’). Over time, these symptoms have an adverse effect on mental health, physical activity and quality of life (QoL). The condition requires innovative and feasible treatment approaches that can be effective and self-managed. Physical activity is essential for good health; however, aerobic exercise or weightlifting may not be suitable for post COVID patients who experience fatigue or breathlessness. The benefits of yoga include improved flexibility, mobility, body strength and balance. It is also shown to reduce symptoms of fatigue and improve breathing efficiency, mental health and QoL. This study protocol describes the rationale and methods for a randomised controlled trial (RCT) of a yoga-based intervention designed for adults with post COVID.

**Methods and analysis:**

A two-group, parallel, RCT with blinded follow-up assessments. Participants will be randomised with a 1:1 allocation to either a 12-week yoga-based intervention or a 12-week health promotion (active comparison) intervention. In total, 88 participants aged 30–65 years will be recruited and randomised. The primary outcome is health-related QoL (36-item Short-Form). Secondary outcomes are dyspnoea, fatigue, sleep quality, cognitive functions, mental fatigue, depression, anxiety, physical activity, demographic data and physical health measures. Data will be analysed as intention-to-treat basis, using linear mixed modelling. All assessments are conducted at Karolinska Institutet in Stockholm, Sweden. The yoga-based intervention will take place at a yoga studio centrally located in Stockholm city.

**Ethics and dissemination:**

The study is approved by the Swedish Ethical Review Authority, reference number 2023/06518-01. All participants must sign written informed consent before enrolment and are free to withdraw from the study at any point. Key results will be available through research articles and seminars.

**Trial registration number:**

German Clinical Trials Register, DRKS00032837.

**STRENGTHS AND LIMITATIONS OF THIS STUDY**

- The condition-specific yoga programme was codesigned by yoga experts from India and adapted for use in Sweden.
- Providing compensation for completing participation and keeping routine follow-ups reduces the risk of attrition bias.
- Giving a similar amount of attention to the control group by health information is reducing the likelihood of a Hawthorne effect.
- The trial lacks a long-term follow-up assessment.

## Introduction

 Post-COVID-19 condition (post COVID, also known as long COVID) is a threat to public health and individual well-being requiring new treatment options. According to the WHO, the condition begins usually within 3 months from the onset of COVID-19, lasts for a minimum of 2 months and cannot be explained by an alternative diagnosis.^1^ Data from The Swedish Board of Social Affairs and Health (Socialstyrelsen) indicated that between October 2020 and March 2022, approximately 25 000 people in Sweden were diagnosed with post COVID.^2^ The intensity of the condition is heterogenous and related to the varying severity of clinical symptoms.^3^ Common symptoms include fatigue, dyspnoea (breathlessness) and cognitive dysfunction (including ‘brain fog’).^4^ Over time, these symptoms have a detrimental effect on mental health, physical activity levels and overall quality of life (QoL).^5 6^

Physical activity is fundamental for health and well-being; however, aerobic exercise and strength training may not be suitable for individuals with post COVID as common symptoms (eg, fatigue and breathlessness) may hinder participation or exacerbate the symptoms.^7^ Yoga is an increasingly popular activity which is potentially more suitable for this specific condition. The ancient Indian practice of yoga refers to a unity of the body, mind and spirit, with a holistic outlook on health and well-being. The physical benefits include improved flexibility, mobility, core body strength and balance.^8^

To date, there is no single intervention shown to effectively reduce the multidimensional symptoms of post COVID. Although the effects of yoga have not been assessed in those with post COVID, research has shown positive effects in many of the symptoms commonly associated with the condition. Yoga has beneficial effects on the autonomic nervous system and immune response, as well as contributing to hormonal changes associated with positive mood states.^9^ Additionally, yoga is shown to improve heart rate variability, which is considered a proxy marker for autonomic nervous system regulation and cardiovascular health.^10^ Decreases in chronic physiological stress through yoga may partly explain the positive effects of yoga on anxiety^11^ and cognition.^12^ Furthermore, recent meta-analyses indicate that yoga practice is associated with improvements in fatigue,^13^,^15^ depression and anxiety^11 16^ and sleep quality^17^ when compared with control conditions. It has been suggested that the health benefits of yoga may be mediated by improved sleep quality.^18^ Experiencing extreme fatigue and impaired sleep can have detrimental effects on cognitive abilities and concentration, and often lead to a reduction in health-related QoL (HR-QoL).^19^ Yoga is also shown to help reduce dyspnoea in chronic obstructive lung disease and related conditions.^20^

Post COVID is an emerging threat to health, estimated to affect more than 100 million people globally.^21^ Yoga, with its interdisciplinary approach of physiological and psychological mechanisms, may prove to be a valuable tool for holistic well-being and recovery in the aftermath of the pandemic.^22 23^

### Rationale

Current approaches to the treatment and management of post COVID are largely symptom based. There is a need to develop lifestyle-based approaches that will improve heterogenous symptoms and can be self-managed and maintained with limited supervision. Yoga has the potential to ease the symptoms and sequelae of post COVID. However, trials are needed to determine its effectiveness. Findings will support clinicians in making evidence-informed treatment decisions. Our study targets middle-aged adults, as this group are disproportionately affected by post COVID and are likely to experience significant morbidity as they age.

To develop a condition-specific yoga programme for post COVID, we have established a collaboration with expert researchers from the Institute of Public Health in Gujarat, India, and the Yoga University, Gujarat. Our international research team includes experts in yoga-based exercise and post COVID research. Through a consensus development process, our expert colleagues have identified postures, awareness practices and breathing techniques most suitable for people with post COVID. The process is described in a forthcoming paper.^24^ These validated methods will be adapted locally in Sweden to optimise the effects of the intervention and minimise adverse events. Concurrently, our Indian collaborators will be conducting a parallel study in India during the same period.

### Study objective

This trial aims to examine the effects of a 12-week yoga intervention on HR-QoL among adults aged 30–65 years diagnosed with post COVID. Results will be compared with a 12-week health promotion control group.

#### Research questions

1. What are the effects of a 12-week yoga intervention compared with a health promotion intervention on HR-QoL in adults aged 30–65 years with post COVID?
2. What are the effects of yoga on secondary health outcomes, including fatigue, sleep quality, dyspnoea, cognition (including ‘brain fog’) and mental health?
3. Is sleep quality a mediating factor in the relationship between yoga practice and HR-QoL?

#### Hypotheses

We hypothesise that (1) compared with a health promotion intervention, participation in a 12-week yoga intervention will be associated with improvements in HR-QoL. We also hypothesise that (2) participation in a 12-week yoga intervention will have positive effects on all secondary health outcomes. Furthermore, we hypothesise that (3) sleep quality mediates the relationship between yoga and improved HR-QoL.

## Methods and analysis

### Trial design

**Figure 1:**
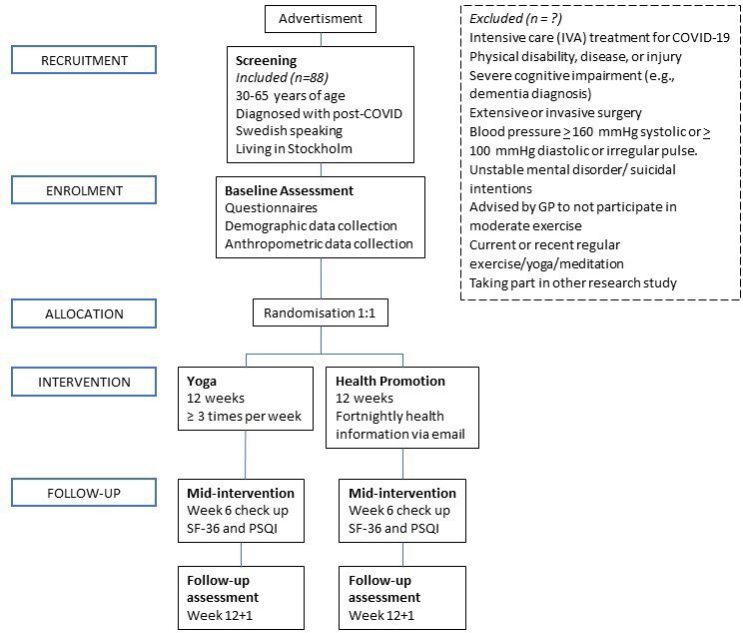
Participant flowchart for parallel design based on the CONSORT guidelines^25^ for transparent reporting of trials. CONSORT, Consolidated Standards of Reporting Trials; GP, general practitioner; PSQI, Pittsburgh sleep quality index; SF-36, 36-item Short Form.

We propose an open-label, two-group parallel randomised controlled trial (RCT) with blinded follow-up assessments (see participant flowchart for parallel design following Consolidated Standards of Reporting Trials guidelines^25^ in figure 1). Participants will be randomised to one of the two conditions: (1) a 12-week supervised yoga programme or (2) a 12-week health promotion (active comparison) intervention. Both interventions are complementary to usual care (ie, ongoing treatment for post COVID recommended or prescribed by a physician). This study protocol follows Standard Protocol Items: Recommendations for Interventional Trials 2013, recommended guidelines for a clinical trial protocol.^26^

### Study setting

The study will take place in Stockholm County. The trial will be conducted at the department of global public health, Karolinska Institutet. The yoga-based intervention will be undertaken at a yoga studio centrally located in Stockholm with a trained yoga instructor.

### Eligibility criteria

A trained research assistant will perform the eligibility screenings. To be eligible for this study, participants must be between 30 and 65 years of age, have residence in Stockholm County, be fluent in Swedish and give consent to randomisation and participation in the trial. The participants must have a diagnosis of post COVID confirmed by their own physician, be able to attend baseline and follow-up assessments at Karolinska Institutet and respond ‘yes’ to the question ‘have your COVID-19-related symptoms had a negative impact on your quality of life?’.

### Exclusion criteria

1. Participants who have received intensive care treatment for COVID-19. Participants treated in emergency departments or primary care facilities remain eligible.
2. Physical disability, disease or injury that could interfere with, or be worsened by, yoga practice (eg, paralysis, inability to sit, stand and/or walk, severe pain, glaucoma).
3. Severe cognitive impairment (eg, dementia diagnosis).
4. Extensive surgery during the past year or planned for the coming year.
5. Raised blood pressure≥160 mm Hg systolic or ≥100 mm Hg diastolic (measured objectively in a sitting position) or irregular pulse.
6. Recently diagnosed with, or affected by, serious mental illness (eg, psychosis or bipolar disorder) or indicating acute signs of suicidality (eg, recent, or ongoing thoughts of suicide or self-harm).
7. Diagnosed with post orthostatic tachycardia syndrome.
8. Pregnancy.
9. Advised not to participate in moderate exercise by their doctor (insulin-dependent diabetes will need doctors’ agreement before participating).
10. Currently or recently engaged in regular structured exercise, or already practicing yoga or meditation.
11. Currently taking part in another research study.

### Interventions

#### Yoga

Participants randomised to the yoga group will receive a free 3-month membership to a registered yoga studio centrally located in Stockholm. The studio will offer group yoga classes designed specifically for this project. The classes will be adjusted to the needs of those with post COVID, including symptoms of fatigue and dyspnoea. Participants will be encouraged to attend ≥3 classes per week with a qualified yoga instructor. After attending onsite classes for the first 2 weeks to familiarise themselves with the programme and the instructor, they will have the option to join classes online on occasions where onsite participation is not an option. Attendance and type of participation are taken for each participant in each class. The length of the intervention is consistent with other yoga-based interventions.^27^ The yoga programme has been developed through a consensus development process by our expert collaborators at the Yoga University, India, and consists of postures and breathing techniques suitable for people with post COVID symptoms.^24^ A short description of the programme, which is broadly based on Hatha and Kundalini yoga principles, is available in online supplemental file 1. Physical postures (asanas) included in the programme are gentle shoulder, arm and neck movements, forward bends, gentle supine and sitting twists, aiming to improve flexibility, mobility and core strength, while also facilitating stress reduction.^8 9^ Additionally, several breathing techniques (pranayamas), such as alternate nostril breathing and humming bee breathing, are included, followed by relaxation and meditation techniques, such as focused attention and body awareness. The duration of a class is between 45 and 60 min and consists of a combination of the physical postures followed by breathing and relaxation techniques. Classes are held mid-morning or mid-afternoon and will be delivered by qualified yoga instructors trained by our expert collaborators in India.

#### Health promotion (control)

Participants randomised to the health promotion group will receive fortnightly updates consisting of health promotion information (also for 3 months). A study-specific health promotion web page will be developed and updated with general health information; topics will include healthy eating, nature therapy, social connectedness, sleep habits and substance use (eg, strategies to quit smoking and reduce alcohol intake). Additional content will address post COVID concerns, providing practical guidance on managing fatigue, dyspnoea and ‘brain fog’. The contents will be concise (1–2 pages) and sourced from reliable organisations, including the WHO, and local health promotion agencies. An example of the health promotion content and format is available in online supplemental file 2. Participants will access the web page through a link sent fortnightly via email. To prevent group contamination, no health promotion information will be shared regarding breathing control, stretching or ‘mindfulness’ practices. Participants in the health promotion group will be advised not to take part in yoga, Pilates, tai chi or similar forms of exercise as well as any form of meditation during the 12-week intervention. Part of the rationale for delivering the health promotion to participants regularly is to reduce the likelihood of a Hawthorne effect, that is, to ensure the participants receive a similar level of attention. As an incentive, participants randomised to the health promotion group will receive a complimentary membership to the yoga studio when they attend their postintervention assessment.

#### Measures to maintain adherence

The yoga studio chosen for the intervention is located close to public transport connections. A research assistant will contact each yoga participant by phone every 2 weeks to discuss any adherence-related issues (eg, transport or motivation). Participants will be encouraged to contact the study research assistant if they experience an adverse event, such as pain or injury, related to yoga. A research assistant will conduct a 6-week mid-intervention follow-up (all participants) to assess adherence and adverse events. Yoga participants will complete a weekly exercise diary and attendance will be recorded at the yoga studio.

### Primary outcome

HR-QoL will be measured by the 36-item Short-Form (SF-36).^28 29^ The SF-36 is widely reported as a subjective measurement of HR-QoL and includes 36 items that measure each of the following dimensions: physical role functioning, bodily pain, general health perception, vitality, social role functioning, emotional role functioning and mental health. The scores range from 0 to 100, where a higher score indicates better HR-QoL.

### Secondary outcomes

1. Dyspnoea will be assessed with the self-rated instrument, the Multidimensional Dyspnea Profile, which assesses sensory qualities of breathlessness, overall breathing discomfort and emotional responses. Each participant will be asked to identify and rate the activity that triggers maximal dyspnoea.^30^
2. Fatigue will be assessed by the self-rating instrument for fatigue, the 9-item Fatigue Severity Scale. The scale provides separate scores for the physical, social and cognitive domains of fatigue, as well as a total combined measure of fatigue.^31^
3. Symptoms of depression and anxiety will be assessed using the Hospital Anxiety and Depression Scale (HADS), which consists of two 7-item subscales. Items are scored between 0 and 3 according to how the respondent felt during the past week.^32^ An advantage of using HADS in the context of post COVID is its avoidance of questions on somatic symptoms, which may conflate reported levels of depression and anxiety.
4. Cognitive function will be measured by using the Trail Making A+B test^33^ and the Verbal Fluency test.^34^ The tests are reliable measures of cognitive flexibility, attention span, visual search and processing speed; all domains discussed to be impaired by post COVID.^35^ Furthermore, the subjective measurement tool, Mental Fatigue Scale, will assess ‘brain fog’, mental fatigue and dysfunction. The scale consists of 15 items assessing general fatigue, stress, sensitivity and sleep.^36^
5. Sleep quality will be assessed with the subjective measurement Pittsburgh Sleep Quality Index (PSQI). The 19-item scale measures seven domains; subjective sleep quality, sleep latency, sleep duration, habitual sleep efficiency, sleep disturbances, use of sleep medication and daytime dysfunction.^37 38^ The seven domains are then combined to a global score of sleep quality.
6. Physical activity will be assessed with the short 4-item version of the International Physical Activity Questionnaire, a subjective self-report on vigorous, moderate and low physical activity.^39^
7. Physical health measures include blood pressure (Omron M3 Comfort), resting heart rate, body mass index, grip strength (Vetek EH101), waist–hip ratio, blood oxygen levels (GIMA Pulse Oximeter OXY-3) after a sit-to-stand test, pulmonary function (COSMED microQuark spirometer) and heart rate variability (Actiheart V.5).
8. Baseline measures will include demographic data of age, gender, education, residential status and employment status, alongside self-reported indicators of somatic health regarding the nature and duration of post COVID symptoms, comorbidities, medications, tobacco use and alcohol consumption.^40^

### Sample size

As there are no published RCTs of yoga for post COVID, the power estimate was based on data from our RCT of yoga-based exercise for well-being among physically inactive older adults,^41^ conducted in the same setting. A sample size of 35 participants per condition (yoga and health promotion intervention) would provide ~90% power (two-tailed α=0.05) to detect an effect size of 0.28 (small) on the primary outcome (SF-36). This equates to a postintervention group difference of ≥5 points (or ≥0.5 SD increase/improvement) on each of the two summary scores (mental and physical components). With an expected dropout rate of 20% and 1:1 allocation, the total number of participants estimated at baseline is 88.

### Recruitment

Adults with post COVID aged 30–65 years will be recruited via an advertisement in a newspaper (Mitt-I) distributed free of charge throughout Stockholm. To support recruitment, a selection of general physicians in the Stockholm County region and a specialist COVID-19 clinic (Karolinska Hospital) will be provided with written information about the trial, which can be passed on to potential participants. The study will be briefly advertised as an opportunity to participate in a 12-week intervention for adults with post COVID. Participation in one of the two activities will be described: yoga or health promotion. Those interested in participating will contact a research assistant who will first conduct a brief screening over telephone, and then arrange a meeting to evaluate eligibility and perform baseline assessments.

### Randomisation and blinding

An independent statistician based at Karolinska Institutet will generate a sex-stratified randomisation list using a random number computer programme. The allocation sequence will be kept confidential and transferred into sealed envelopes by an administrator not involved in the study. Envelopes will be opened by the participants immediately following baseline assessments. Group allocation will be recorded on separate forms that are not available to follow-up assessors. Participants will be instructed not to discuss group allocation during follow-up appointments when a research assistant blinded to group allocation will be collecting data. Allocation will be kept confidential and only unblinded in the event of participant withdrawal or adverse events leading to ending participation.

### Data collection

#### Baseline assessment

The baseline assessment will be performed by trained research assistants prior to allocation. The assessment covers demographic data and the collection of primary and secondary outcomes, including anthropometric measurements, questionnaires and objective measurements (see Outcomes section). Baseline assessments are scheduled to take 60–90 min and are conducted daytime with a maximum difference of 3 hours. All questionnaire data will be collected using the REDCap software hosted on secure university servers requiring two-step authentication.^42 43^

#### Follow-up assessments

**Figure 2:**
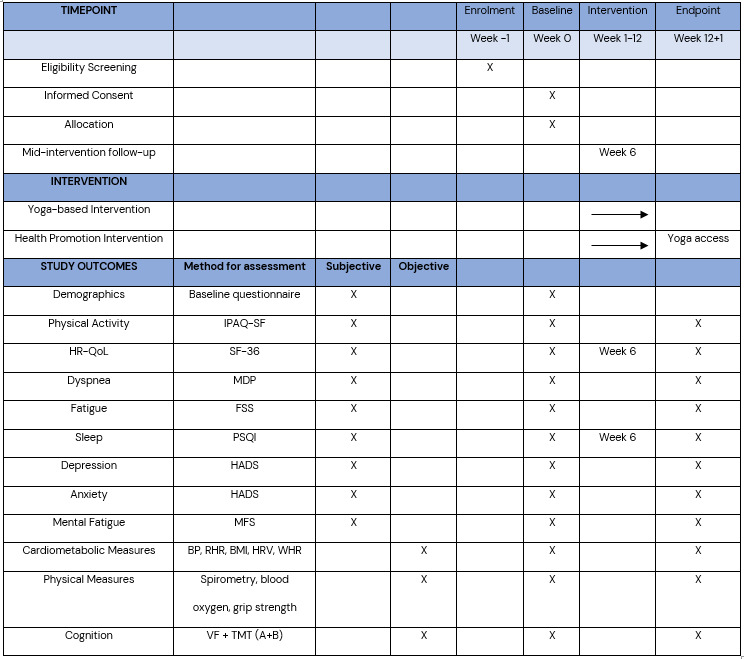
Schedule of enrolment, interventions and assessments, according to SPIRIT guidelines.^26^ BMI, body mass index; BP, blood pressure; FSS, Fatigue Severity Scale; HADS, Hospital Anxiety and Depression Scale; HR-QoL, health-related quality of life; HRV, heart rate variability; IPAQ-SF, International Physical Activity Questionnaire Short Form; MDP, Multidimensional Dyspnea Profile; MFS, Mental Fatigue Scale; PSQI, Pittsburgh Sleep Quality Index; RHR, resting heart rate; SF-36, 36-item Short Form Health Survey; SPIRIT, Standard Protocol Items: Recommendations for Interventional Trials; TMT A+B, Trail Making test A+B; VF, Verbal Fluency; WHR, waist–hip ratio.

All participants will be followed up in person at half-time (6 weeks) to discuss progress, adherence and occurrence of any adverse events associated with the intervention. Assessments at 6 weeks will include the SF-36 and the PSQI. Postintervention assessments of all primary and secondary outcomes will be performed at 12+1 weeks, following the same procedure as at baseline (see figure 2). A brief semistructured interview is held at post intervention to gather participant views on the intervention. Postintervention assessments are held at the same time as baseline assessment for each participant.

### Data management

Study participants will receive a unique study ID at inclusion. Collected data will be coded and kept confidential in participant case report forms using the same ID, which is considered source data. Source data will be transferred into electronic form and combined with questionnaire data collected in REDCap. Collected data will be handled only by authorised people and kept in a locked archive at the department of global public health, Karolinska Institute. The principal investigator is responsible for the maintenance and security of project data. A senior researcher not otherwise involved in the project will read the standard operating procedures and assess whether data collection and management have been followed correctly at 6 and 12 weeks.

### Statistical methods

A statistician not otherwise involved in the project will perform the analyses. The study collects quantitative data and intention-to-treat analyses will be performed to examine the effects of the yoga-based intervention compared with the health promotion. Missing data will be replaced using multiple imputations. Effects of the intervention will be explored using linear mixed modelling, where group-by-time interactions will be reported with regression coefficient estimates, CIs and effect sizes (Hedge’s g). As a secondary analysis, we will explore sleep as a potential mediator of the effects of yoga on HR-QoL. Sensitivity analyses will be performed, comparing imputed and non-imputed datasets. As ‘contamination’ effects are possible (eg, if health promotion participants decide to engage in yoga at their initiative), an ‘as treated’ analysis will be undertaken. Moderating effects of sex and age will be examined, as well as interaction effects of education on cognitive outcomes. All analyses will be carried out at study endpoint when data collection is completed.

### Assessment of harms

Trained research assistants will screen all potential participants and exclude those with health-related problems that might hinder participation or be worsened by yoga. Throughout the study, all adverse events will be reported, evaluated and followed up by a research assistant. Any adverse events needing medical attention will be addressed and referred to appropriate medical professionals. Adverse events will be formally assessed at 6-week follow-up and at post intervention (12+1 weeks) by asking participants if they had experienced any worsening of symptoms, injuries or other adverse efforts during the trial. Participants who report adverse events will be advised to see their general practitioner for further evaluation. All participants will be instructed to contact the study coordinator if any adverse events occur during the intervention. Decisions of participant or study discontinuation based on adverse effects will be made by the principal investigator. Participants can decide to dropout from the study at any given time. The yoga instructors are certified by the Yoga Alliance Guidelines and trained in minimising the risk of injuries and harm.

### Patient and public involvement

During the development of this trial, we contacted the Swedish COVID-19 Association and the largest COVID-19 treatment clinic to obtain their views regarding the inclusion and exclusion criteria proposed in our study design. Based on their feedback, some minor changes to these criteria were made. With the exception of this important element of the study, we did not directly involve the public in other aspects of the study design. The research questions and measures included in the trial were chosen by a multidisciplinary team of researchers that includes specialist physicians involved in the treatment of post COVID patients.

### Ethics and dissemination

The trial is approved by the Swedish Ethical Review Authority (Etikprövningsmyndigheten), number 2023/06518-01. All participants will be informed about the aims and procedure of the study prior to participation. Written informed consent will be obtained by a research assistant prior to enrolment in the study from all participants (see consent form in online supplemental file 3) Confidentiality, voluntariness and freedom to withdraw participation at any time will be stated. Participants will give written informed consent to include their non-identified data in aggregate analyses that will be published in a scientific journal. No identifying or personal details of participants will be presented in any form. The findings of the study will be published in leading scientific journals and presented at conferences and seminars to key stakeholders. We have plans to disseminate the findings back to the Swedish COVID Association and a selection of COVID-19 clinics and health centres. A summary of key results will be published for participants of the study and the general population through newspaper articles, press releases and media channels.

## Discussion

Post COVID remains prevalent, yet there is no agreed treatment regime due to the heterogenous profile of the condition; consequently, many clinicians struggle to provide effective treatments. This RCT aims to broaden the array of potentially effective treatment options by examining the effects on HR-QoL of a yoga-based intervention, designed specifically for adults diagnosed with post COVID. The findings will provide new insights into the effects of a lifestyle-based intervention that can be self-maintained beyond the 12-week programme. The intervention is likely to improve the health and well-being of those with post COVID symptoms and has the potential to be incorporated into clinical practice (eg, by referring those with mild-to-moderate symptoms to appropriately trained yoga practitioners). Further, it may encourage research on lifestyle treatments for related chronic conditions, or research on yoga’s effect on physiological mechanisms (eg, why pulmonary function may be enhanced by yoga, how yoga practice affects inflammatory biomarkers, how metabolism is affected, etc). Trials have been conducted using specific components of yoga in post COVID context, such as breathing techniques,^44^ meditation techniques for cognition^45^ or online-based yoga for psychological well-being.^46^ This is the first RCT to investigate the effects of a complete yoga programme specifically designed for adults with post COVID on HR-QoL and secondary health outcomes.

Post COVID can be difficult to diagnose, being dynamic and multisymptomatic, and an individualised treatment plan may take time to initiate. Symptomatic treatment varies on a patient-to-patient basis and may include pharmacotherapy or rehabilitation.^47^ Most medical treatments provide only partial symptom relief, and many have side effects, resulting in poor compliance. Rehabilitative treatments include cognitive behavioural therapy for mental health and fatigue,^48 49^ physical therapy for pain^50^ and pulmonary rehabilitation.^51^ A comprehensive approach to a patient’s recovery often involves several treatments.

Yoga is a holistic treatment option for reducing allostatic load in post COVID.^23^ Yoga practice is discussed to have anti-inflammatory effects,^52^ and a proposed mechanism behind this may be enhanced melatonin production.^53^ Recent studies indicate that yoga may have a beneficial effect on the diversity and composition of the gut microbiome, which may reduce fatigue and improve immunity and mental health.^54^ Further, practicing yoga has been associated with positive effects on blood circulation and brain health.^55 56^ By stimulating the parasympathetic nervous system and the vagus nerve, yoga practice regulates the hypothalamic–pituitary–adrenal axis and improves heart rate variability,^10 57 58^ which is shown to be an indicator of cardiovascular and general health. Additionally, isometric poses have been associated with a reduction in fatigue and increased vitality in chronic fatigue syndrome, a condition sharing features with post COVID.^14^ Few treatment options offer integrated health benefits to the same extent as yoga practice.

Yoga-based exercise is a non-stigmatising, readily available and relatively inexpensive option, which could complement traditional medical care, potentially reducing the need for medication while improving HR-QoL. Yoga can be practised almost anywhere, guided by instructors onsite or digitally, and adjusted to meet the individual’s needs. Our recent collaborations with researchers in India have shown that yoga is a safe and feasible activity for adults with no previous experience^41 59 60^ and associated with fewer adverse events than moderate-intensity aerobic exercise.^61^

There are some unique challenges associated with this trial. Given that fatigue is a common symptom among patients with post COVID, some participants may find it challenging to complete the intervention, potentially leading to higher than predicted attrition rates. To reduce the risk of attrition bias, fortnightly phone calls to the yoga participants will be conducted, during which adherence and motivational issues will be discussed with a research assistant trained in cognitive psychology. Offering yoga memberships to participants in the health promotion group (following completion of the trial) serves as an incentive to complete the trial. Another potential challenge is the scheduling of classes to avoid conflicts with work or other commitments. To overcome this, classes will be offered at various times on weekdays, with the option to attend online on occasions when it is inconvenient to travel to the studio. However, participants will be encouraged to join onsite classes to gain the most benefit from the programme, especially during the first few weeks of the trial. A potential limitation is that the study lacks long-term follow-up, and thus, maintenance of intervention effects cannot be examined. Findings from the trial will be used to support grant applications for studies involving long-term assessments (12 months) and mechanistic effects of yoga in post COVID.

### Trial status

Protocol version 3 of 2024-08-14. Recruitment is planned to begin in the end of January 2024 with a total of three recruitment periods planned (spring 2024, fall 2024 and spring 2025). Data collection is estimated to be completed in May 2025.

## supplementary material

10.1136/bmjopen-2024-085525 online supplemental file 1

10.1136/bmjopen-2024-085525 online supplemental file 2

10.1136/bmjopen-2024-085525 online supplemental file 3
