# Supplementary material for: Effects of yoga compared with health promotion on health-related quality of life in adults with post-COVID-19 condition: protocol for a randomised controlled trial
Source: BMJ Open. 2024 Sep 12;14(9):e085525. doi: 10.1136/bmjopen-2024-085525 (PMC11404200; doi:10.1136/bmjopen-2024-085525)
Supplement: online supplemental file 1 [file bmjopen-14-9-s001.pdf]

YOGA MODULE FOR LONG COVID – Short Version

Table of Content

1. Part A – Sukshma Vyayama (gentle movements)

The first section consists of gentle movements to warm up the neck, shoulders, and upper body.

2. Part B – Simple breathing exercises

Second section consists of simple breathing exercises which are focusing on simple repetitive movements in sync with inhalation and exhalation.

3. Part C – Asanas (physical postures)

Asanas are physical postures practiced for promoting physical and mental health. The poses range from simple and relaxing to challenging and static. Asanas target specific muscle groups, joints, or organs, aiming to enhance flexibility, strength, balance, and body awareness. They can be addressed separately or in a flow, often guided by one's breathing.

4. Part D – Pranayama (breathing techniques)

Pranayama is the yogic practice of breath control, involving various techniques. Through conscious regulation of inhalation, exhalation, and breath retention, pranayama aims to optimize the flow of *prana* (i.e., life force energy) throughout the body.

5. Part E – Relaxation techniques

Yoga relaxation techniques including a variety of practices aimed at promoting physical and mental calmness. Methods mentioned here include guided imagery, muscle relaxation, and deep relaxation. Relaxation techniques is best practiced guided by an instructor or teacher.

How to make use of this module

A sequence/full practice created based on this module will contain one or two items from each part in the following order:

- Loosening up (by gentle movements, gentle stretching)
- Simple breathing exercises
- Asanas (either a few separate ones, or in a structured flow with your breath)
- Deep relaxation (for example savasana)
- Pranayama (end the practice with pranayama)

The level of difficulty can be adjusted based on abilities and capacity of the individual participant, and many combinations can be created. A more detailed description of each posture or exercise are available upon request.

Part A – Sukshma Vyayama

1. Greeva Sanchalana (Neck Movements)

Gentle clockwise and anticlockwise head rotations with full awareness. Warms up neck, and includes physical benefits like neck mobility, tension relief, and improved posture.

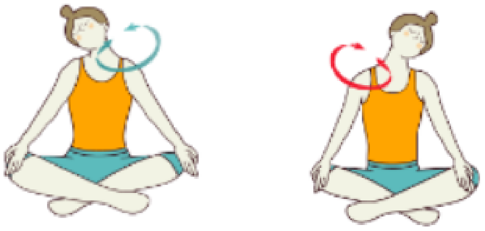

2. Skanda Sanchalana (Shoulder Movements)

Slow clockwise and anticlockwise rotations of the shoulders. Warms up arms, shoulders, and shoulder blades. Includes physical benefits like improved blood circulation, stimulates lung capacity, and the cardiovascular system.

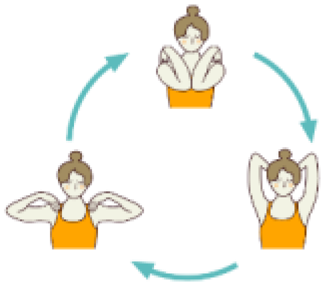

Part B – Breathing Exercises

1. Hands In and Out Breathing

This exercise brings awareness to the body, increases breathing and lung capacity.

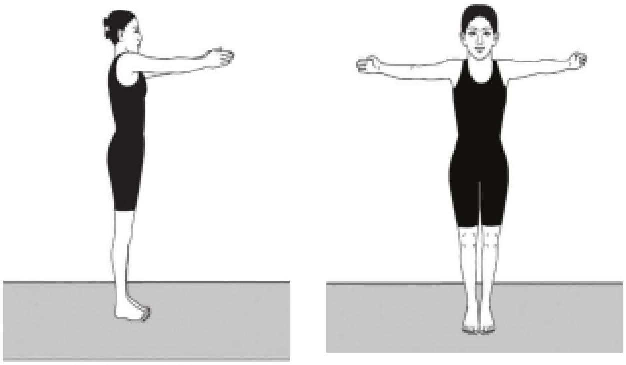

2. Hand Stretching Breathing

This motion can be alternated by either stretching horizontally, slightly upward at forehead level or straight above head. This exercise brings awareness to the body, increases breathing and lung capacity.

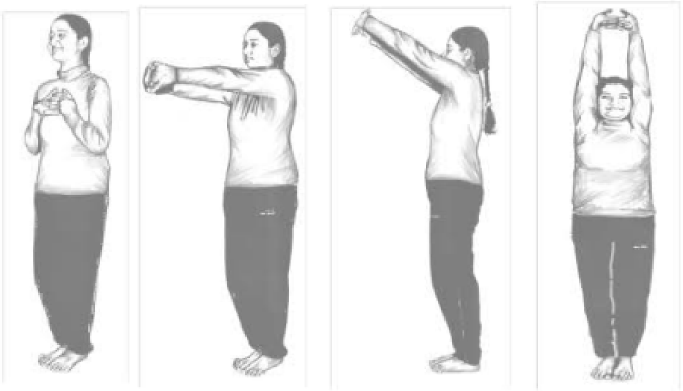

3. Tiger Breathing

This "all-fours" exercise warms up the core, and stimulates glands and organs of abdominal area, digestion and improves circulation of spinal fluid.

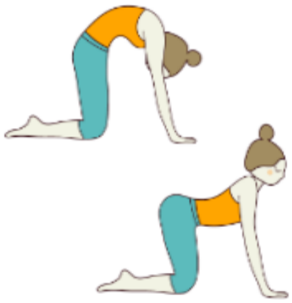

4. Shashankasana Breathing

This kneeling exercise increases blood circulation and blood flow to the brain, relieves lower back pain and strengthens joints of feet and ankles.

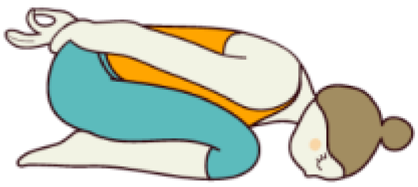

5. Straight Leg Raising

This supine exercise can be done by alternating raising right or left leg, or raising both legs, depending on core strength. This exercise strengthens abdominal and core muscles, relieves lower back pain, and improves blood circulation.

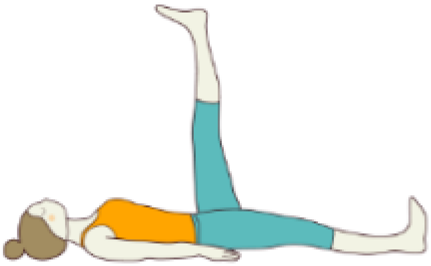

6. Chair Surya Namaskar (Sun Salutation)

This seated flowing exercise strengthens the torso, expands chest, and improves lung capacity and digestion.

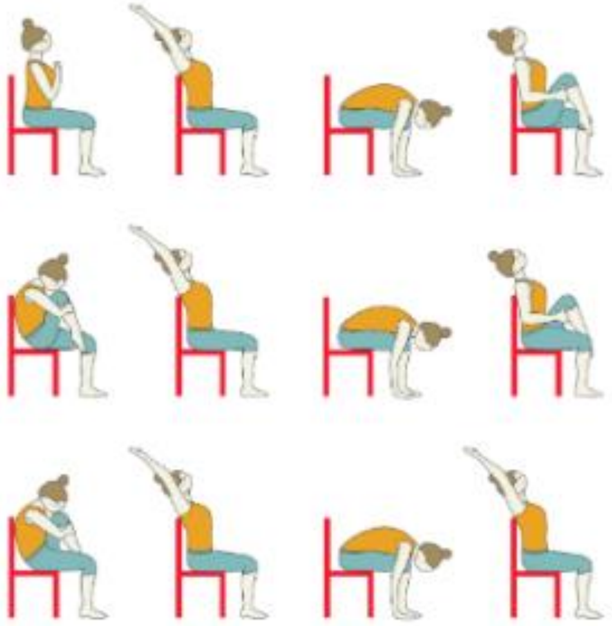

Part C – Asanas

1. Ardha Chakrasana (half-wheel pose)

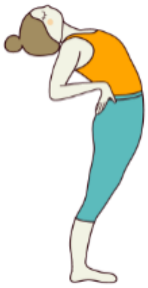

Standing pose, which stimulates internal organs, regulates blood pressure, increases lung capacity, and improves balance.

2. Ardhakati Chakrasana (half-waist wheel pose)

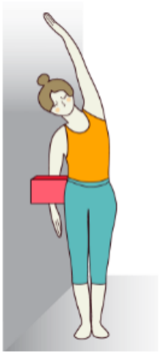

Standing pose, which improves respiratory function, stimulates internal organs, strengthens spine, and improves balance.

3. Marjariasana (tiger pose)

"All-fours" pose, which strengthens spine and core, improves digestion, and stimulates glands and organs of the abdominal area.

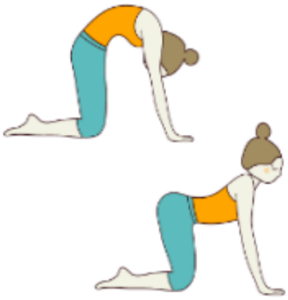

4. Sarala Matsyendrasana (simple sage matsyendra's pose/lord of fishes' pose)

Sitting pose, which stretches and open chest and airways, increases lung capacity, stimulates internal organs, and improves blood circulation.

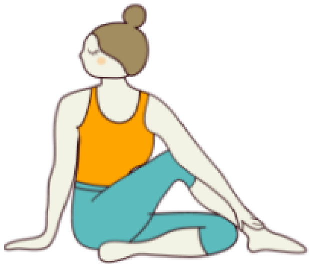

5. Trikonasana (triangle pose)

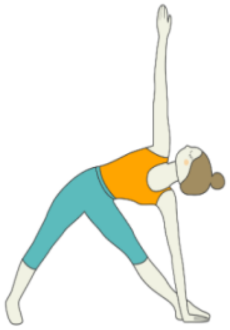

Standing pose, which expands chest and lengthens spine, increases lung capacity, and improves balance.

6. Ustrasana (camel pose)

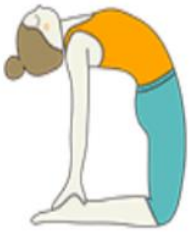

Kneeling pose, which opens chest, strengthens spine and neck.

7. Bhujangasana (cobra pose)

Prone pose, which opens chest, creates expansion in chest and rib cage, and improves digestion.

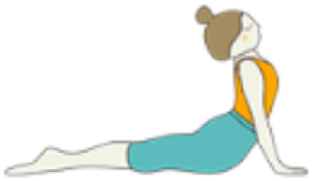

8. Savasana (corpse pose)

Supine pose, which calms the nervous system, improves respiratory and cardiovascular function.

9. Setubandhasana (bridge pose)

Supine pose, which strengthens the back and core, increases blood circulation, and stimulates urogenital organs.

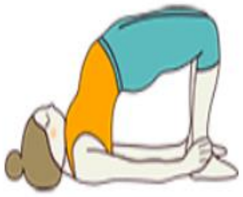

10. Pavanamuktasana (wind-relieving pose)

Supine pose, which relieves lower back pain and improves blood circulation.

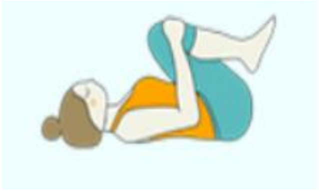

11. Sarala Matsyasana (simple fish pose)

Supine pose, which opens chest, improves respiratory and cardiac systems, stimulates lymphatic system, and improves digestion.

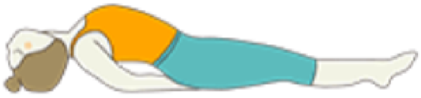

Part D – Pranayama

1. Nadishuddi Pranayama (alternate nostril breathing)

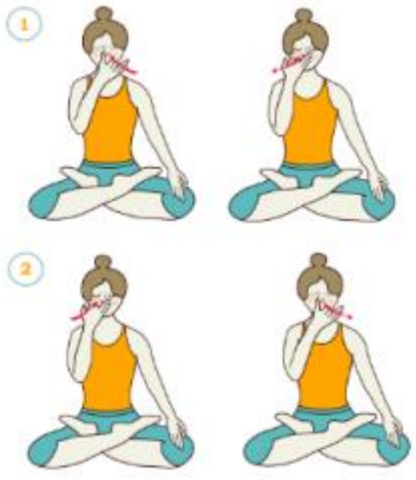

2. Bhramari Pranayama (humming bee breath)

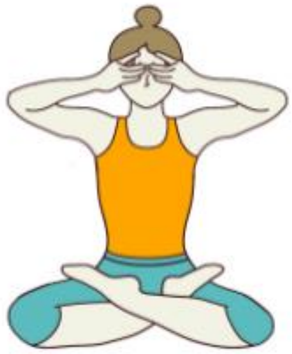

3. Ujjayi Pranayama (ocean breath/victorious breath)

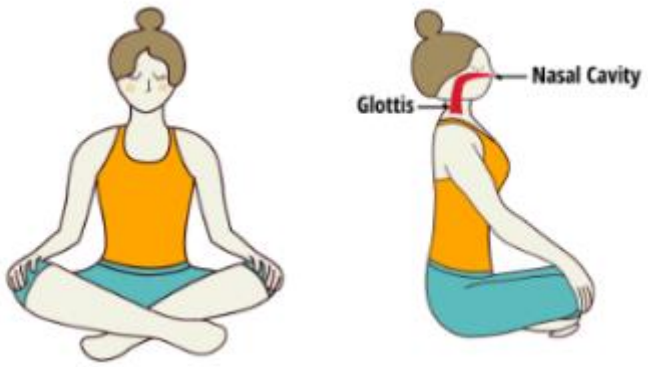

4. Vibhagiya Svasa (sectional breathing)

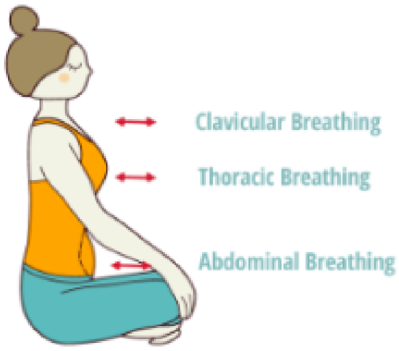

5. Nadanusandhana Pranayama (meditating on sound)

Part E – Yogic Relaxation Techniques

1. Yoga Nidra (yogic sleep technique)
2. Instant Relaxation Technique (IRT)
3. Quick Relaxation Technique (QRT)
4. Deep Relaxation Technique (DRT)
5. Mind Sound Resonance Technique (MSRT)
6. Cyclic Meditation (CM)
7. OM Meditation
