# Supplementary material for: Effects of yoga compared with health promotion on health-related quality of life in adults with post-COVID-19 condition: protocol for a randomised controlled trial
Source: BMJ Open. 2024 Sep 12;14(9):e085525. doi: 10.1136/bmjopen-2024-085525 (PMC11404200; doi:10.1136/bmjopen-2024-085525)
Supplement: online supplemental file 2 [file bmjopen-14-9-s002.pdf]

Health Promotion Module: Dyspnea

Dyspnea is a common symptom of post COVID, referring to a self-experienced sensation of breathing difficulties and shortness of breath. Dyspnea consists of multiple aspects and sensations that can vary in intensity, leading to negative effects on daily functioning. These aspects can involve the nature of breathing, its intensity, the degree of discomfort, and the emotional consequences. This symptom often results in a vicious cycle of decreased activity, reduced fitness, and increased discomfort. It is associated with depressive symptoms, anxiety, and reduced quality of life. There are treatments available to alleviate dyspnea, although the underlying cause can be difficult to define. Depending on the specific cause and how it is experienced, the treatment methods may vary. Provided that your dyspnea is not caused by serious cardiovascular problems, it is important to stay as active as you can.

A few tips on how to ease dyspnea:

1. Raise your upper body with the help of a few extra pillows when you sleep or rest. This can help open the airways. Cool air may also make breathing easier, so ensure your room is at a comfortable cool temperature. It may be helpful to have a small fan on hand.
2. Stay hydrated throughout the day, and drink plenty of water. Dehydration can worsen dyspnea.
3. Take frequent, short breaks when performing a task that may trigger your dyspnea, such as climbing stairs or going outside in chilly, damp weather. Let each movement or activity take as much time as it needs.
4. Keep a diary of your breathing experience. When does it feel okay, and when is the discomfort increasing? How does it make you feel? By mapping out and identifying what may trigger your dyspnea, you will gain a better understanding of the symptom and finding ways to manage it can be easier.
5. Anxiety and sense of panic can worsen dyspnea in a vicious cycle. Try to remain calm by finding methods to soothe your mind and, eventually, your body. Perhaps a phone call to a loved one, a bath, listening to calming music, or physical touch like a hug or a gentle stroke on the back, may help.

You can read more about dyspnea and tips on how to ease your breathing through the links below:

Dyspné

Andningsbefrämjande åtgärder
