# Supplementary material for: Effects of yoga compared with health promotion on health-related quality of life in adults with post-COVID-19 condition: protocol for a randomised controlled trial
Source: BMJ Open. 2024 Sep 12;14(9):e085525. doi: 10.1136/bmjopen-2024-085525 (PMC11404200; doi:10.1136/bmjopen-2024-085525)
Supplement: online supplemental file 3 [file bmjopen-14-9-s003.pdf]

Participant Consent Form

Please read the information sheet carefully and do not hesitate to ask questions

- ☐ I have received information regarding the study's purpose and procedures and have been given the opportunity to ask questions.
- ☐ I have been informed that my participation is entirely voluntary
- ☐ I have been informed that I can choose to end my participation at any time during the study
- ☐ I have received sufficient oral and written information regarding the study
- ☐ I consent to my personal data being recorded as described above and handled only by authorized personnel involved in the study
- ☐ I consent to my data being included in aggregated analyses for publication in scientific journals

I consent to participate in the study

---	------------------
Signature	Place and date
---	------------------
Printed name	Birthdate (YYMMDD)

The undersigned has informed the above-mentioned participant of the study's purpose and procedures and obtained their signed informed consent. The participant has received a copy of the study information sheet.

---	------------------
Signature of research assistant or equivalent	Place and date

Printed name
