# Supplementary material for: Effects of yoga compared with health promotion on health-related quality of life in adults with post-COVID-19 condition: protocol for a randomised controlled trial
Source: BMJ Open. 2024 Sep 12;14(9):e085525. doi: 10.1136/bmjopen-2024-085525 (PMC11404200; doi:10.1136/bmjopen-2024-085525)
Supplement: Supplementary file 4 [file bmjopen-2024-085525.reviewer_comments.pdf]

PEER REVIEW HISTORY

BMJ Open publishes all reviews undertaken for accepted manuscripts. Reviewers are asked to complete a checklist review form and are provided with free text boxes to elaborate on their assessment. These free text comments are reproduced below.

ARTICLE DETAILS

**TITLE (PROVISIONAL)**	Effects of yoga compared with health promotion on health-related quality of life in adults with post COVID-19 condition: protocol for a randomized controlled trial
**AUTHORS**	Brodén, Mikaela; Welfordsson, Paul; Niemi, Maria; Diwan, Vinod; shah, komal; Pattanadara, Vijayakumar; Hallgren, Mats

VERSION 1 - REVIEW

**REVIEWER NAME**	Bhandari, Rudra
**REVIEWER AFFILIATION**	University of Patanjali
**REVIEWER CONFLICT OF INTEREST**	I declare no competing interest.
**DATE REVIEW RETURNED**	12-Mar-2024

**GENERAL COMMENTS**	After thoroughly going through the proposed RCT on the effects of yoga on health-related quality of life in adults with post-COVID-19 conditions, I found it interesting and relevant. However, the below comments are over to address:<br>1. What is meant by post-COVID? Does it indicate the after-effects of clinically significant COVID-19 infection?<br>2. Measuring subjective and objective indices has nothing to do with the risk of bias in RCTs. See point 3 of the article summary on page 3.<br>3. What is the rationale for sampling adults aged between 35-65 years?<br>4. "The intervention will be undertaken at a local yoga facility; please see line 34 on page 7. Clarify.<br>5. The intensity of post-COVID conditions may be related to the varying severity of clinical symptoms of COVID-19. Clarify under inclusion criteria. Online and offline options to go through intervention may induce heterogeneous impacts. Mention controlling techniques for the same.<br>6. Cite techniques of yoga practices included under intervention.<br>7. The option of uncertain timings for intervention may induce different effects and contraindications.<br>8. Health promotion protocol is unclear.<br>9. What is meant by a medical outcomes study? See line 36 on page 10.<br>10. Conduct a pilot study or refer to a published RCT to compute the effect size and justify the sample size. Refer to https://doi.org/10.1177/09727531221117623. The cited effect size is irrelevant and related to the effect of yoga-based exercise on the well-being of physically inactive older adults.

	11. Baseline measurement time points seem different participant-wise. This may threaten the internal validity of the study.<br>12. How can over nine outcome measures be taken within 60 minutes?<br>13. The claim of this study, being the first RCT to investigate the effect of yoga on post-COVID symptoms under discussion, is untrue. Refer to the study https://doi.org/10.1177/09727531221117623<br>14. Cite 2nd paragraph contents of the discussion.<br>15. Please see under trial status: “Recruitment is planning to begin at the end of January 2024 and is estimated to be completed in May 2025.” This shows a 17-month duration only for participant recruitments, which may create serious variation in the timings and dates of baseline measurements.<br>16. Scan grammatical mistakes throughout the manuscript.<br>17. It is unusual to cite submitted papers. See reference 21.

**REVIEWER NAME**	Boaventura, Paula
**REVIEWER AFFILIATION**	IPATIMUP
**REVIEWER CONFLICT OF INTEREST**	None.
**DATE REVIEW RETURNED**	18-Apr-2024

**GENERAL COMMENTS**	General Comments<br><br>The study (protocol) addresses a very pertinent and interesting issue. It is well designed and well written. I have only some small comments which I believe should be addressed.<br><br>Specific Comments<br><br>1. Page 4, Line 12 – The authors state “First RCT to investigate the effects of yoga on post COVID symptoms.” I think this is not completely true. In a brief research I found other RCTs: “Safety and efficacy of Ayurvedic interventions and Yoga on long term effects of COVID-19: A structured summary of a study protocol for a randomized controlled trial” Yadav et al., 2021; “Efficacy of pursed lip breathing with bhastrika pranayama vs incentive spirometry in rehabilitating post covid 19 follow up-a randomized control study”.<br><br>2. Page 5, Line 11 – Substitute “three months” with “usually three months”, as stated in the WHO definition.<br><br>3. Page 5, line 34 – “may hinder participation” – In fact is may even aggravate their state an also may aggravate their state: https://www.clinicalkey.com/#!/content/playContent/1-s2.0-S0025619623001763?scrollTo=%23bib27 Mueller et al., 2023<br><br>4. Page 6, Line 31 – I believe this paper, an overview of yoga for COVID, should be included here: Yoga for COVID-19: An ancient practice for a new condition – A literature review Capela Santos et al, 2023.<br><br>5. Page 6, Lines 35-54 - I think this could be better written, or summarized, it is a bit repeated.<br><br>6. Page 9, Line 57 - Will the presences be counted? How many sessions will the participants have to attend to be included in the

	evaluation? Why did the authors allow on-line classes at the beginning of the study for participants who may never have done yoga before? My suggestion is that in this first 12 week approach, online yoga should not be included. This could be an approach for patients who have ended the intervention, so they can pursue their self care.<br><br>7. Page 17, Line 43 – Again, this is not correct. See comment 1.<br><br>8. Page 17, Line 55 – This is not clear. Which are the treatments that are effective, and which are the ones that are partially effective?<br><br>9. Page 17, Line 59 – "cognitive behavioural therapy" is not indicated for CFS/ME, so I use it in the post-COVID condition?<br><br>10. Page 18, Line 8 – The paper mentioned in comment 4, should also be included here.<br><br>11. Page 18, Line 56 - As a suggestion: why not leave 5 minutes at the end of class to discuss participant's feelings and issues eventually arising? This is common in yoga sessions in east countries. In our own experience (a pilot study we are conducting in Portugal), restorative yoga is very well tolerated by these patients.

VERSION 1 – AUTHOR RESPONSE

Reviewer 1.

Dr. Rudra Bhandari, University of Patanjali

1.1. What is meant by post-COVID? Does it indicate the after-effects of clinically significant COVID-19 infection?

Authors response: Thank you for the question on post COVID. Post COVID is a diagnosis of long-lasting debilitating symptoms after COVID-19, also known as long COVID or post-COVID-19-condition. According to World Health Organization definition, the condition begins usually within three months from the onset of COVID-19, lasts for a minimum of two months and cannot be explained by an alternative diagnosis. Post COVID is a multisymptomatic condition, and the most common symptoms are fatigue, dyspnea, 'brain fog', and somatic pain. Other symptoms that may occur are digestive issues, tinnitus, headaches, fever, perception sensitivity and cardiovascular issues such as hypertension or post orthostatic tachycardic syndrome (POTS). Over time, these symptoms have a detrimental effect on mental health, physical activity levels, and overall quality of life. Post COVID-19 condition is described in the protocol in the main text in 'Introduction', see page 4, lines 2-12.

1.2. Measuring subjective and objective indices has nothing to do with the risk of bias in RCTs. See point 3 of the article summaries on page 3.

Authors response: Thank you, we have removed this point from the summary. Page 3, lines 14-15.

1.3. What is the rationale for sampling adults aged between 35-65 years?

Authors response: The main reason for focusing on middle-aged adults is that they are the group most affected by post COVID, so we wanted to target the largest population. Other factors that also influenced the decision were: the risk of cognitive deficiency and age-related comorbidities increases markedly after the age of 65, which could potentially complicate the analyses; the 30-65 age group may still be relatively responsive to lifestyle interventions and may have a greater capacity to adopt and sustain new habits compared to older adults who experience greater multimorbidity (including musculoskeletal problems). We have added to the manuscript: “Our study targets middle-aged adults as this group are disproportionately affected by post COVID and are likely to experience significant morbidity as they age”. See page 5, lines 24-25.

1.4. “The intervention will be undertaken at a local yoga facility; please see line 34 on page 7. Clarify.

Authors response: The yoga-based intervention is held at a yoga studio centrally located in Stockholm, with access to public transport. We have clarified in the protocol, see page 7, lines 18-19.

1.5.1 The intensity of post-COVID conditions may be related to the varying severity of clinical symptoms of COVID-19. Clarify under inclusion criteria.

Authors response: Thank you for your comment. We will exclude those who were admitted to intensive care for treatment of post COVID symptoms for these reasons: (1) to reduce the heterogeneity of symptom severity in the study sample, and (2) to eliminate the risks associated with yoga for those with e.g., severe lung damage or other serious health complications. Please see exclusion criterion listed on page 8, lines 4-6. We have added in the Introduction main text: “The intensity of the condition is heterogenous and related to the varying severity of clinical symptoms”. See page 4, lines 8-9.

1.5.2 Online and offline options to go through intervention may induce heterogeneous impacts. Mention controlling techniques for the same.

Authors response: Offering online classes is necessary for optimizing adherence, as many participants are working full- or halftime and may have long commute to the yoga studio. Some may experience symptoms that limit how often or far they can travel. The online classes are identical to the classes held in the studio, as they are live streamed from the yoga studio. We control for this by recording attendance on type of participation for each class, if the participant has joined online or onsite. This has now been stated in the Intervention subsection: “Attendance of type of participation are taken for each participant each class”. See page 9, lines 8-9.

1.6. Cite techniques of yoga practices included under intervention.

Authors response: Thank you for your comment. A brief description of the techniques (physical postures and breathing exercises) is presented in the manuscript on page 9. Complete details of the program (all techniques used) are available as supplementary material (this is noted in the manuscript). We have added suitable references to yoga practices and that the yoga program is broadly based on Hatha and Kundalini yoga principles, see page 9, line 12, 13, and 16.

1.7. The option of uncertain timings for intervention may induce different effects and contraindications.

Authors response: Thank you for the opportunity to respond. By ‘timings’ we assume that the expert reviewer refers to the intervention being held at different times of the day (mid-morning and mid-afternoon). Classes are only four hours apart, so the different timings are minimal. No early morning or late evening classes are offered. In this way, we have minimized any potential contraindications. To optimize adherence, it is necessary to offer a somewhat flexible schedule. Please see clarifying on page 9, line 21.

1.8. Health promotion protocol is unclear.

Authors response: Could the expert reviewer be more specific by noting exactly what is unclear about this section of the paper? Our description of the comparison group is clear in our opinion; however, we will address any specific concerns.

1.9. What is meant by a medical outcomes study? See line 36 on page 10.

Authors response: Thank you for your question. The Medical Outcomes Study (MOS) was a longitudinal research project in the US, that aimed to understand patient’s quality of life and health outcomes between late 1980s and early 1990s. The MOS contributed to the development of the tool SF-36 to measure health-related quality of life. To avoid uncertainties, this has been removed from the manuscript. Page 10, line 24.

1.10. Conduct a pilot study or refer to a published RCT to compute the effect size and justify the sample size. Refer to https://doi.org/10.1177/09727531221117623. The cited effect size is irrelevant and related to the effect of yoga-based exercise on the well-being of physically inactive older adults.

Authors response: Thank you for your comment. When we performed the original power estimate, there were no studies on which to base the calculation; thus, we used our previous trial (FitForAge) because the context is the same and the intervention is very similar. In 2023-24, three new trials of yoga for COVID-19 patients have been published, but these involve hospitalized patients with severe symptoms and/or different outcome measures (one study did assess HR-QoL, but the authors used a different measure to ours, and the trial was exploratory: https://www.ncbi.nlm.nih.gov/pmc/articles/PMC10282501/. Thus, similar concerns could be raised with the revised power calculation, as these newer studies also differ in important ways from our planned study. In our view, the current estimate is accurate, despite some notable differences in the populations being examined. If the expert reviewer disagrees, we would greatly appreciate the opportunity to revise the power estimate and would appreciate a recommendation regarding which trial to select for the estimate, thank you.

1.11. Baseline measurement time points seem different participant-wise. This may threaten the internal validity of the study.

Authors response: The difference is minimal; baseline assessments are conducted at 10AM or 1PM (just 3 hours apart). The participants are followed up at the same time as their baseline assessments. This is now clarified in the manuscript, see page 14, line 3; page 14, lines 12-13.

1.12. How can over nine outcome measures be taken within 60 minutes?

Authors response: We appreciate the reviewer's comment on this matter and agree that all outcomes in 60 minutes is an optimistic estimate. 60 minutes was the original time frame, however in practice it takes 60-90 minutes to complete all measurements. This has now been revised in the protocol to 60-90 minutes. See page 14, line 3.

1.13. The claim of this study, being the first RCT to investigate the effect of yoga on post-COVID symptoms under discussion, is untrue. Refer to the study https://doi.org/10.1177/09727531221117623

Authors response: We acknowledge that this is not the first trial to investigate yoga for post COVID-19 condition. It is however the first study to investigate the effect of a complete yoga program specifically designed for non-hospitalized patients with post COVID-19 condition, that includes the following three components (1) physical postures (asanas); (2) breathing techniques (pranayamas) and (3) relaxation techniques. The unique features of the trial have been clarified as follows: "Trials have been conducted utilizing specific components of yoga in post COVID context, such as breathing techniques (43), meditation techniques for cognition (44), or online-based yoga for psychological well-being (45). This is the first randomized controlled trial to investigate the effects of a complete yoga program specifically designed for adults with post COVID on health-related quality of life and secondary health outcomes". Please see page 18, lines 3-8.

1.14. Cite 2nd paragraph contents of the discussion.

Authors response: Thank you for this comment regarding references of the discussion. We have revised the 2nd paragraph of the discussion and added references of statements regarding treatment options. Please see page 18, lines 11-16.

1.15. Please see under trial status: "Recruitment is planned to begin at the end of January 2024 and is estimated to be completed in May 2025." This shows a 17-month duration only for participant recruitments, which may create serious variation in the timings and dates of baseline measurements.

Authors response: Recruitment is divided in three periods, with commencement in January 2024, August 2024, and January 2025. Recruitment is ongoing for one month, with instant start of the intervention after the baseline assessment. There are minor variations in timings and dates of baseline assessments, however the follow-up assessments are conducted at the same time during the day as baseline assessments for the individual participant. We acknowledge the expert's point about the duration of the timing, but recruitment is challenging in this population, and that is the time realistically needed to meet our target. We have revised the 'Trial Status' subsection and made the following change: "Recruitment is planned to begin in the end of January 2024 with a total of three recruitment periods planned (spring 2024, fall 2024, and spring 2025). Data collection is estimated to be completed in May 2025". See page 20, lines 10-11.

1.16. Scan grammatical mistakes throughout the manuscript.

Authors response: We appreciate the encouragement from the reviewer and will ensure to proof-read the manuscript carefully before re-submitting.

1.17. It is unusual to cite submitted papers. See reference 21.

Authors response: Thank you for your comment. We choose to retain the reference for the submitted paper for the reason of it being of importance in the context of the consensus development process behind the yoga program. Hopefully, this key paper will be accepted during the revision of this protocol.

Reviewer 2.

Dr. Paula Boaventura, IPATIMUP

2.1. Page 4, Line 12 – The authors state “First RCT to investigate the effects of yoga on post COVID symptoms.” I think this is not completely true. In brief research I found other RCTs: “Safety and efficacy of Ayurvedic interventions and Yoga on long term effects of COVID-19: A structured summary of a study protocol for a randomized controlled trial” Yadav et al., 2021; “Efficacy of pursed lip breathing with bhastrika pranayama vs incentive spirometry in rehabilitating post covid 19 follow up-a randomized control study”.

Authors response: We acknowledge that this is not the first trial to investigate yoga for post COVID-19 condition. It is however the first study to investigate the effect of a complete yoga program specifically designed for non-hospitalized patients with post COVID-19 condition, that includes the following three components (1) physical postures (asanas); (2) breathing techniques (pranayamas) and (3) relaxation techniques. The unique features of the trial have been clarified as follows: “Trials have been conducted utilizing specific components of yoga in post COVID context, such as breathing techniques (43), meditation techniques for cognition (44), or online-based yoga for psychological well-being (45). This is the first randomized controlled trial to investigate the effects of a complete yoga program specifically designed for adults with post COVID on health-related quality of life and secondary health outcomes”. Please see page 18, lines 3-8.

The paper by Yadav et al. (2021) is a protocol; there are still no results.

2.2. Page 5, Line 11 – Substitute “three months” with “usually three months”, as stated in the WHO definition.

Authors response: We agree with the comment and have adjusted the description of the WHO definition accordingly. See page 4, line 4.

2.3. Page 5, line 34 – “may hinder participation” – In fact is may even aggravate their state an also may aggravate their state: https://www.clinicalkey.com/#!/content/playContent/1-s2.0-S0025619623001763?scrollTo=%23bib27 Mueller et al., 2023

Authors response: We thank the reviewer for the comment and insight on physical activity with post COVID, and that symptoms may be worsened. We have elaborated in the revised manuscript with a suitable reference. See page 4, line 15.

2.4. Page 6, Line 31 – I believe this paper, an overview of yoga for COVID, should be included here: Yoga for COVID-19: An ancient practice for a new condition – A literature review Capela Santos et al, 2023.

Authors response: Thank you, we agree with the reviewers' comment and have added the reference. See page 5, line 14.

2.5. Page 6, Lines 35-54 - I think this could be better written, or summarized, it is a bit repeated.

Authors response: Thank you, we have revised the 'Rationale' subsection as follows; "Current approaches to the treatment and management of post COVID are largely symptom-based. There is a need to develop lifestyle-based approaches that will improve heterogenous symptoms and can be self-managed with limited supervision. Yoga has the potential to ease the symptoms and sequalae of post COVID. However, trials are needed to determine its effectiveness. Findings will support clinicians in making evidence-informed treatment decisions". See page 5, lines 15-25.

2.6. Page 9, Line 57 - Will the presences be counted? How many sessions will the participants have to attend to be included in the evaluation? Why did the authors allow on-line classes at the beginning of the study for participants who may never have done yoga before? My suggestion is that in this first 12-week approach, online yoga should not be included. This could be an approach for patients who have ended the intervention, so they can pursue their self-care.

Authors response: We thank the reviewer for the comments and would like to respond as follows: (1) Attendance is recorded by the yoga instructor onsite. Attendance is continuously followed-up by a research assistant, who has regular (fortnightly) meetings with the yoga instructor. Attendance is also discussed during pre-arranged phone calls and meetings with the participants at 2 weeks, 6 weeks (half time) and 9 weeks. Additionally, the participants fill out activity calendars which are handed in at the end of the intervention. (2) Number of attendances required: As the primary analyses are intention to treat (ITT), no minimum number of attendances are required. For as treated analyses, we will adopt specific criteria (>= 12 sessions in total). (3) Online classes: The option to participate online is included for feasibility reasons and to enhance attendance and is only possible after the participant have been onsite for the first two weeks, so they get to know the program and the instructor. The online classes are streamed live from the yoga studio. Following the first recruitment period (just ended), we see that most participants have chosen onsite yoga. See amendments of adherence and online options on page 9, lines 5-9.

2.7. Page 17, Line 43 – Again, this is not correct. See comment 1.

Authors response: We acknowledge that this is not the first trial to investigate yoga for post COVID-19 condition. It is however the first study to investigate the effect of a complete yoga program specifically designed for non-hospitalized patients with post COVID-19 condition, that includes the following three components (1) physical postures (asanas); (2) breathing techniques (pranayamas) and (3) relaxation techniques. The unique features of the trial have been clarified as follows: "Trials have been conducted utilizing specific components of yoga in post COVID context, such as breathing techniques (43), meditation techniques for cognition (44), or online-based yoga for psychological well-being (45). This is the first randomized controlled trial to investigate the effects of a complete yoga program specifically designed for adults with post COVID on health-related quality of life and secondary health outcomes". See page 18, lines 3-8.

The paper by Yadav et al. (2021) is a protocol; there are still no results.

2.8. Page 17, Line 55 – This is not clear. Which are the treatments that are effective, and which are the ones that are partially effective?

Authors response: Thank you for your question(s). A review of all the treatment options and their effectiveness is beyond the scope of our study protocol. Which treatments are effective or partially effective depends on the individual participant, the severity and diversity of their symptoms and other co-existing treatments. Post COVID is a multisymptomatic condition and patients seldomly experience only one or two symptoms. Patients may receive medication for pain, which is partially effective in terms of relieving pain intensity however not curing or relieving other symptoms associated with post COVID such as fatigue or dyspnea. Most treatments are symptom based, often pharmaceutical, and have side effects. We have revised the paragraph in the manuscript. See full 2nd paragraph of the Discussion on page 18, lines 9-17.

2.9. Page 17, Line 59 – “cognitive behavioural therapy” is not indicated for CFS/ME, so I use it in the post-COVID condition?

Authors response: We agree that CBT is not recommended as a priority treatment for CFS/ME, however, the method may support patients in managing some of their symptoms, and studies do support this. In the context of post COVID, CBT shows promising effects for aiding severe fatigue, insomnia, and mental health. We acknowledge that the use of CBT may be viewed as an inappropriate substitute for other effective treatments; its use in patients with CFS/ME appears to divide both researchers and many clinicians. See amendments in the manuscript on page 18, line 15 (references 48 and 49).

2.10. Page 18, Line 8 – The paper mentioned in comment 4, should also be included here.

Authors response: Thank you, we have added the reference here. See page 18, line 18.

2.11. Page 18, Line 56 - As a suggestion: why not leave 5 minutes at the end of class to discuss participant’s feelings and issues eventually arising? This is common in yoga sessions in east countries. In our own experience (a pilot study we are conducting in Portugal), restorative yoga is very well tolerated by these patients.

Authors response: Thank you for this suggestion. At the end of each class, most participants are relaxed and in a mindful state which we do not wish to disturb. Also, participants may not wish to discuss feelings and experiences in a group (privacy issues) and discussing with each participant individually would not be feasible. The yoga instructor is not a counsellor nor a physician, so we do not believe this would be appropriate. Participant views on the intervention are systematically gathered at the follow-up assessment using a brief semi-structured interview. We have added the following statement in the manuscript: “A brief semi-structured interview is held at post-intervention to collect participant views on the intervention”. See page 14, line 11-12.

VERSION 2 - REVIEW

**REVIEWER NAME**	Bhandari, Rudra
**REVIEWER AFFILIATION**	University of Patanjali
**REVIEWER CONFLICT OF INTEREST**	No.
**DATE REVIEW RETURNED**	16-Jul-2024

**GENERAL COMMENTS**	Health promotion protocol should be detailed like the yoga protocol.

**REVIEWER NAME**	Boaventura, Paula
**REVIEWER AFFILIATION**	IPATIMUP
**REVIEWER CONFLICT OF INTEREST**	None
**DATE REVIEW RETURNED**	18-Jun-2024

**GENERAL COMMENTS**	The authors have carefully adressed all my comments.

VERSION 2 – AUTHOR RESPONSE

Reviewer 1.

Dr. Rudra Bhandari, University of Patanjali

Specific Comment

1.1. Health Promotion should be detailed like the yoga protocol

Authors response: Thank you for your comment. We have refined the subsection on Health Promotion to clarify the content and distribution. Additionally, an example of the health promotion content format of the dyspnea module is available as supplementary material. Please see pages 9-10 for changes in main text, and supplementary file 2 for content format.

Reviewer 2.

Dr. Paula Boaventura, IPATIMUP

Specific Comment

2.1. The authors have carefully addressed all my comments.

Authors response: Thank you for your comments on our manuscript, we appreciate the time and effort put into reviewing the manuscript.
